# Linking soil fungi to bacterial community assembly in arid ecosystems

**DOI:** 10.1002/imt2.2

**Published:** 2022-02-24

**Authors:** Shuo Jiao, Haiyan Chu, Baogang Zhang, Xiaorong Wei, Weimin Chen, Gehong Wei

**Affiliations:** ^1^ State Key Laboratory of Crop Stress Biology in Arid Areas, Shaanxi Key Laboratory of Agricultural and Environmental Microbiology, College of Life Sciences Northwest A&F University Yangling China; ^2^ State Key Laboratory of Soil and Sustainable Agriculture, Institute of Soil Science Chinese Academy of Sciences Nanjing China; ^3^ State Key Laboratory of Subtropical Silviculture Zhejiang A&F University Hangzhou China; ^4^ State Key Laboratory of Soil Erosion and Dryland Farming on the Loess Plateau Northwest A&F University Yangling Shaanxi China

**Keywords:** arid ecosystems, bacteria, biogeography, biotic factors, community assembly

## Abstract

Revealing the roles of biotic factors in driving community assembly, which is crucial for the understanding of biodiversity and ecosystem functions, is a fundamental but infrequently investigated subject in microbial ecology. Here, combining a cross‐biome observational study with an experimental microcosm study, we provided evidence to reveal the major roles of biotic factors (i.e., soil fungi and cross‐kingdom species associations) in determining soil bacterial biogeography and community assembly in complex terrestrial ecosystems of the arid regions of northwest China. The results showed that the soil fungal richness mediates the balance of assembly processes of bacterial communities, and stochastic assembly processes decreased with increasing fungal richness. Our results further suggest that the predicted increase in aridity conditions due to climate change will reduce bacterial α‐diversity, particularly in desert soils and subsurface layer, and induce more negative species associations. Together, our study represents a significant advance in linking soil fungi to the mechanisms underlying bacterial biogeographic patterns and community assembly in arid ecosystems under climate aridity and land‐use change scenarios.

## Highlights

- Soil fungal richness mediates the bacterial community assembly.
- Stochastic assembly processes decreased with increasing fungal richness.
- Increase in aridity reduced bacterial α‐diversity.

## Graphical abstract

Our findings revealed the major roles of biotic factors and aridity in shaping the bacterial landscapes and mediating the community assembly in complex terrestrial ecosystems. Soil fungal richness mediates the balance of assembly processes of soil bacterial communities, with stochastic assembly processes decreasing along with an increase in fungal richness. Increased aridity conditions due to climate change could reduce the bacterial α‐diversity, with more negative effects in desert soils and subsurface layers, and induce more antagonistic or competitive biological interactions.

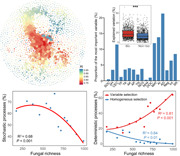

## INTRODUCTION

Soil microbes play important roles in a variety of ecological processes in terrestrial ecosystems, including soil decomposition, nutrient cycling, pollutant degradation, and maintaining stable ecosystem services in face of environmental changes [1, 2, 3]. Revealing the fundamental mechanisms that underpin microbial community diversity and biogeographic patterns is crucial for determining their linkage with community stability and ecosystem functions, which are key topics in community ecology [4, 5, 6, 7]. The viewpoint in microbial biogeography, everything is everywhere, but the environment selects [8], highlights the remarkable dispersal ability and niche fitness of microorganisms [9, 10, 11]. Yet, it is widely acknowledged that both deterministic and stochastic processes influence the biogeographic patterns of microbial communities and distance‐decay relationships (DDRs) (i.e., microbial community similarity decreases as geographical distance increases) [2, 12]. Deterministic processes involve nonrandom and niche‐based mechanisms [13], including environmental filtering and interspecific interactions (e.g., competition, facilitation, mutualisms, and predation). In contrast, stochastic processes mainly reflect random changes in the relative abundance of species, involving random birth, death, and dispersal events [14, 15]. Several microbial biogeographic studies across various habitats [12, 16, 17] and different scales (e.g. regional [12, 17, 18], continental [10], and global [19, 20, 21]) are turning attention toward the importance of quantifying the contributions of the two major processes that drive microbial community assembly, which is still an ongoing debate [22]. Currently, characterizing the entire range of processes underpinning spatial variation in microbial communities across complex terrestrial ecosystems remains a challenge [23, 24, 25].

Within the framework of deterministic and stochastic processes, previous research has focused on investigating how abiotic factors (e.g., edaphic, climatic, and geographic) affect microbial community structure across habitats with a wide range of environmental heterogeneity [26]. For example, environmental factors, such as soil pH, nutrients, soil texture, and climatic conditions can significantly affect microbial community distribution [27, 28, 29]. However, far fewer studies explore the roles of biotic interactions between microbial taxa in shaping community assembly, which could determine the functional attributes or niche occupancy of microbial communities [30, 31, 32]. For example, species interactions due to fitness differences, for example, competition and mutualisms, could result in the niche partitioning of community members under environmental heterogeneity [33, 34]. This could be inferred based on the species co‐occurrence patterns and their network topological properties [35, 36, 37]. It is assumed that the highly unexplained variation in microbial β‐diversity is attributed to the large varieties of species co‐occurrence patterns and topological features in microbial networks across different spatial scales [26, 38, 39].

Elucidating the factors that mediate the balance between stochastic and deterministic processes could advance the mechanistic understanding of community assembly processes [7, 22, 40, 41]. For example, variation in soil pH could influence the assembly processes that shape soil bacterial communities during pedogenic processes [42]; sulfur can mediate the balance between stochastic and deterministic processes of agro‐soil fungal communities, as indicated by greater stochasticity found at higher sulfur concentration [43]. Particularly, interactions between fungi and bacteria are common in soils [30, 44, 45, 46], which play important roles in stimulating ecosystem processes [47]. For example, soil bacteria and fungi could share common resources, and competition for substrate might induce the antagonism between bacteria and fungi [48]; soil fungi may dominate the decomposition of the recalcitrant organic matter, for example, lignin, and bacteria may symbiotically utilize the fungal‐derived substrates [49].

In the present study, we used a cross‐biome observational field study and a controlled microcosm experiment to test the influence of biotic factors on the bacterial community assembly. We first conducted a soil analysis of 251 samples along the Hexi Corridor, which is a representative oasis–desert ecotone in the arid regions of northwest China [50]. Specifically, we explored the biogeographic patterns of cross‐biome soil bacterial communities at large spatial scales and quantified the contributions of abiotic and biotic factors (i.e., soil fungi and cross‐kingdom species associations) on bacterial β‐diversity and community assembly. We also used a controlled microcosm experiment to examine the effect of fungal richness on the assembly processes of the soil bacterial community. Here, we hypothesize that (i) biotic interactions between microbial taxa contribute to substantial variations in community β‐diversity across different habitats at large scales; (ii) soil fungi mediate the balance of assembly processes of soil bacterial communities. Our study could provide a perspective on the important roles of biotic factors in shaping the bacterial landscapes and community assembly in complex terrestrial ecosystems, which should not be overlooked under land‐use change scenarios.

## METHODS

### Field survey

#### Soil sampling

**Figure 1:**
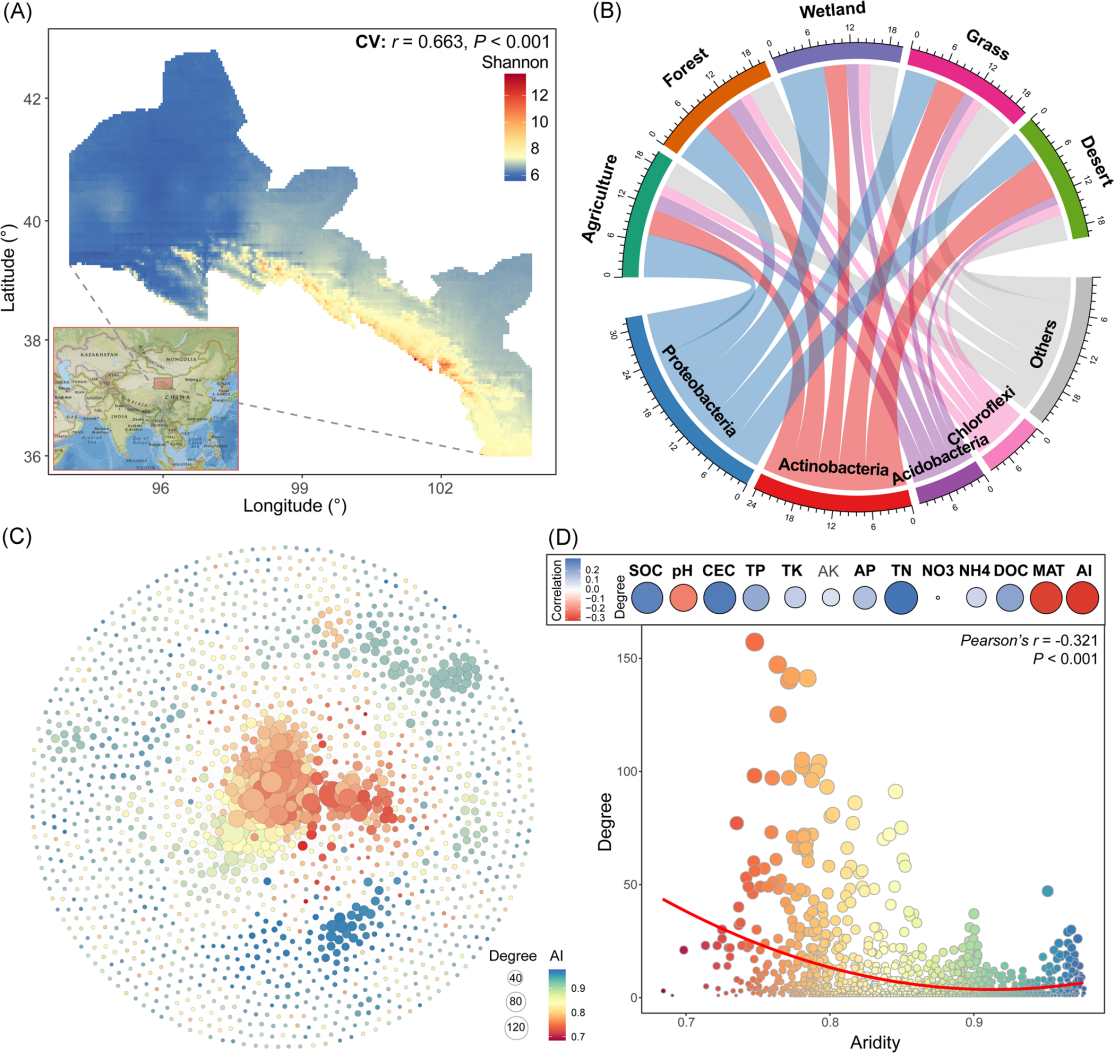
General patterns of soil bacterial diversity and co‐occurrence network. (A) Predicted spatial distribution of bacterial α‐diversity (Shannon index) using the co‐kriging interpolation method. (B) A circos plot showing the taxonomic distribution of soil bacterial taxa among different biomes at the phylum level. The thickness of each ribbon represents the relative abundance of bacterial taxa assigned to different phyla. (C) Cross‐kingdom co‐occurrence network of microbial taxa. The networks are colored based on the aridity preferences of the taxa. A connection indicates a strong and significant (*p* < 0.001) correlation, divided into positive (Spearman's *p* > 0.6; dark gray) or negative (Spearman's *p* < −0.6; red) edges. The size of each node is proportional to the degree of the ASVs; the thickness of a connection between two nodes (i.e., an edge) is proportional to the value of Spearman's correlation coefficient. (D) Correlations between the degree of the microbial taxa within the ecological network and their environmental preferences (top panels). The linear relationships between the degree of the microbial taxa and the environmental preference of aridity (AI) (bottom panels). ASV, amplicon sequence variant

One hundred and twenty‐six sites were selected, 37 in agricultural field, 28 in forest, 15 in wetland, 26 in grassland, and 20 in desert. The sampling sites extended from 36°56' N to 40°34' N, and 94°37' E to 103°31' E (transect intervals of 1257.6 km) along the Hexi Corridor in the northwest of China (Figure 1). The dominant species in these habitats included *Zea mays* (agricultural field), *Calligonum* spp., *Stipa* spp., *Leymus* spp., and *Achnatherum* spp. (wetland, grassland, and desert), and *Populus* spp. (forest). The dominant soil types were Aripsamment and Calciorthids, which have a loose structure and low organic matter content. In July–August 2017 (near the period of the highest aboveground plant biomass), three 100 m^2^ plots were sampled at each site. Five soil cores (2.5 cm diameter) were combined per plot and were taken at depths of 0−15 and 15–30 cm. One subsurface desert sample was abandoned due to DNA extraction failure. Therefore, a total of 251 soil samples were used for this study.

#### Soil properties

Standard methods were used to measure soil pH, moisture, cation exchange capacity (CEC), organic carbon (SOC), dissolved organic carbon (DOC), total nitrogen (TN), nitrate‐nitrogen (NO_3_), ammonium‐nitrogen (NH_4_), total phosphorus (TP), available phosphorus (AP), total potassium (TK), available potassium (AK) as previously described [36, 51]. We obtained climatic data including mean annual temperature (MAT) for all sampling sites from the Worldclim database (www.worldclim.org). In addition, we estimated the aridity (AI, 1–precipitation/evapotranspiration) at each site using the Global Potential Evapotranspiration database [52], which is based on interpolations provided by WorldClim [53].

#### Diversity measures

Soil bacterial and fungal communities were analyzed using high‐throughput amplicon sequencing [54]. Total genomic DNA was extracted from soil samples using a FastDNA SPIN Kit for Soil (MP Biochemicals). The microbial communities were profiled by targeting the V4–V5 region of the 16S rRNA gene for bacteria, and the ITS1 region of the 18S rRNA gene for fungi. The target sequences were amplified by PCR using the primer pairs 515F/907R (bacteria) and ITS5‐1737F/ITS2‐2043R (fungi) [55, 56]. Sequencing was conducted on an Illumina HiSeq. 2500 platform (Illumina Inc.). We assembled quality‐filtered reads into amplicon sequence variants (ASVs) using DADA2 v1.14 [57]. ASVs were filtered when they were present in fewer than two samples. Taxonomy was assigned for sequence identification using The Ribosomal Database Project Classifier tool, implemented using DADA2 accessing the SILVA database (release 138) for bacteria and UNITE + INSD (UNITE and the International Nucleotide Sequence Databases) for fungi [57]. Before we calculated the soil microbial diversity, the ASV tables were resampled to a minimum number of sequences from each sample, at 28,955 for bacteria and 20,065 for fungi. We calculated the Shannon diversity for bacteria and richness for fungi, which were the most extensively used. On average, fungal communities were dominated by Ascomycota (relative abundance: 68.8%), Basidiomycota (10.8%), Mortierellomycota (9.2%), and Chytridiomycota (5.8%) in this order. The raw sequencing data of the field survey study were deposited in the Genome Sequence Archive at the BIG Data Center under BioProject ID PRJCA004036.

### Microcosm study

#### Study site and soil sampling

This microcosm study was conducted in soils independent from the large‐scale survey presented above, explaining the slight methodological differences between these two studies, and enabled us to test relationships between soil fungal richness and bacterial community assembly independently of the data used to assess the spatial patterns. Soil sampling was performed in August 2019 at the location of the arid area in northwest China (38°18' N, 100°9' E). Soil samples were collected at depth of 0–15 cm. The location was a grassland dominated by *Calligonum* spp., and was selected owing to its similar aridity condition (aridity = 0.663) with the sites of the field survey.

#### Microcosm preparation and diversity measurement

Soil samples were sieved to <2 mm to remove the stones and debris, and placed at the artificial climate room (25°C) for several weeks to reach a balanced and stable state. The prepared soil samples were transferred into the plastic pots and amended with fungicide (Cycloheximide) solution to achieve a final concentration of 0 (D0), 6 mg kg^−1^ (D1), and 14 mg kg^−1^ (D2), according to the method in a previous study [58]. The concentration of fungicides was set based on the standard ecotoxicological practice for establishing possible environmental effects of pesticides [59]. A total of 12 microcosms (500 g each; 3 treatments × 4 replicates) were prepared. The moisture contents in these microcosms were adjusted to 60% water holding capacity to allow microbial activities to be maintained (by adding sterile water if needed) during the incubation period. These microcosms were covered with aluminum foil with several holes (1 mm) to avoid contamination and transferred into an artificial climatic chamber. Soil microcosms were incubated at 20°C in the dark for 60 days.

After the incubation, soil samples were collected to conduct diversity measurements, and soil bacterial and fungal communities were analyzed as described above for the cross‐biome study. Before we calculated the soil microbial diversity, the ASV tables were resampled to a minimum number of sequences from each sample, at 55,975 for bacteria and 26,157 for fungi. We calculated the Shannon diversity for bacteria and richness for fungi from rarefied ASV tables. The raw sequencing data of the microcosm study were deposited in the Genome Sequence Archive at the BIG Data Center under BioProject ID PRJCA004037.

### Data analysis

#### Spatial mapping of bacterial diversity

To predict the distribution of the bacterial Shannon diversity across our sampling regions, we applied a co‐kriging interpolation method that takes account of the influence of environmental variables. Here, five environmental predictors were included in the kriging model: soil properties (soil C and pH), climate (AI and MAT), because high‐resolution information on these variables is available at the global scale. The information on soil properties for this grid was obtained using the ISRIC (global gridded soil information) Soil Grids (https://soilgrids.org/#!/?layer=geonode:taxnwrb_250m). This analysis was performed using the automap package [60] in R, which automates the interpolation process by automatically estimating a semivariogram and performing kriging. The cross‐validation of the maps was based on Pearson's correlation between the predicted and observed values in each sampling site using autoKrige.cv in the automap package [60].

#### Correlation networks

To estimate species coexistence across different habitats and regions, cross‐kingdom co‐occurrence networks consisting of bacterial and fungal taxa were constructed. To reduce rare ASVs in the data set, we focused on the microbial taxa that are present in more than 10% of all soil samples. Robust correlations with Spearman's correlation coefficients (*p*) > 0.6 or <−0.6 and *p* < 0.001 were used to construct networks, which has been extensively used in the literature and is comparable across studies [61]. We calculated the average values of each edaphic and climatic factor for sites in which it was detected, which were then weighted by the relative abundance of that taxon per site. These were considered the environmental conditions preferred by each taxon, akin to niche space. The Pearson's correlations were estimated between the environmental preferences' values and the degrees of the nodes.

In addition, we extracted sub‐networks by preserving the phylotypes of individual soil samples using the induced_subgraph function in igraph package in R [62]. The topological features of the sub‐networks in each sample, including average degree (AD), the proportion of negative associations (Neg), and the proportion of interacted associations between bacterial and fungal taxa (Int), were calculated to estimate the potential biotic interactions, which were regarded as biotic factors in examining their contribution to the variation in bacteria α‐ and β‐diversity [26]. The average degree referred to species connectivity in the community [26, 63, 64]. The proportion of negative associations and the proportion of interacted associations between bacterial and fungal taxa could reflect their potential biological interactions [46, 65, 66, 67]. Networks were visualized using the interactive Gephi platform (https://gephi.org).

#### DDRs

DDRs were calculated as the slopes of ordinary least‐squares regressions for the relationships between geographic distances and community similarities (1−dissimilarity of the Bray–Curtis metric). Standard and partial Mantel tests were performed to evaluate the influence of environmental, biotic, and geographic variables upon bacterial community structures, using the vegan package for R [68].

#### Null model analysis

Null model analysis was carried out using the framework described by Stegen et al. [69] to classify community pairs into underlying drivers of deterministic and stochastic processes. The variation in phylogenetic or taxonomic diversity was measured respectively using null‐model‐based phylogenetic β‐diversity metrics (*β*NTI). A neighbor‐joining (NJ) phylogenetic tree was inferred with bootstrap analysis (100 replicates) using the phangorn package [70]. Detailed descriptions of these can be found in previous studies [22, 40, 71]. Briefly, a βNTI < –2 indicates significantly less phylogenetic turnover than expected (i.e., homogeneous selection); conversely, a *β*NTI > 2 indicates significantly more phylogenetic turnover than expected (i.e., variable selection). |βNTI| < 2 indicates the dominance of stochastic processes. We then explored the major factors that influenced the assembly processes of soil bacterial communities. Variation in community assembly processes along the gradients of the derived variables was assessed using the Mantel tests that correlated the βNTI values with the Euclidean distance matrices of each variable. The statistical significance of those comparisons was determined using 999 permutations and the analyses were carried out using the mantel function of the vegan package for R [68].

#### Statistical analysis

We applied the random forest analysis (rfPermute function in rfPermute package in R [72]) and the multiple regression model (lm function in stats package in R [73]) with variance decomposition analysis (calc.relimp function in the relaimpo package in R [74]) to estimate the importance of influencing factors for the topological features. To test the significance and importance of the environmental variables for β‐diversity, we used a distance‐based linear model and forward selection procedure based on the Bray–Curtis distance matrix by estimating the proportion of variance explained (*R* ^2^). These results were displayed by Canonical principal coordinate (CAP) analysis. These analyses were performed using the ordiR2step and capscale function of the vegan package [68]. Standard and partial Mantel tests were performed to evaluate the influence of environmental, biotic, and geographic variables upon bacterial community structures, using the mantel function of the ecodist package for R [75]. The nonmetric multidimensional scaling (NMDS) analysis was performed to visualize the sample relationships across different groups, using the metaMDS function of the vegan package [68].

## RESULTS

### Soil bacterial *α*‐diversity patterns along the Hexi Corridor

Across 251 cross‐biomes soil samples along the Hexi Corridor (transect intervals of 1257.6 km), we modeled the spatial distributions of the soil bacterial α‐diversity (Shannon index; Figure 1A). The bacterial α‐diversity showed a greater value in low latitude and high longitude fields. We observed that most of the sequences belonged to the phyla Proteobacteria, Actinobacteria, Acidobacteria and Chloroflexi, and so forth, which showed different cross‐biome distributions (Figure 1B). Proteobacteria were more abundant in wetland soils, while the relative abundance of Acidobacteria was significantly higher in agricultural and forest soils (Figure S1). Desert soils harbored a relatively higher abundance of Actinobacteria.

We then explored the species colored the higher abundance of the soil microbiota via establishing cross‐kingdom co‐occurrence networks based on correlation, including soil bacterial and fungal taxa. The network consisted of 1740 nodes (i.e., ASVs) and 7088 edges (Figure 1C). We calculated the average values of edaphic and climatic variables for sites in which it was detected; these were considered the environmental conditions preferred by each taxon, akin to niche space. We found that the preference for aridity (AI) showed the highest correlations (Pearson's *r* = 0.321, *p* < 0.001) with the degrees of the nodes (Figure 1D), and the degrees were significantly decreased with an increase in AI (Figure 1D). This suggested closer interconnections and more frequent co‐occurrence among microbial taxa in the lower AI environments. Subsequently, we calculated the topological features of the extracted sub‐networks by preserving the nodes of individual soil samples. Average degree (AD), proportion of negative associations (Neg), and proportion of interacted associations between bacterial and fungal taxa (Int) were calculated to infer the biotic factors. The random forest (Figure S2A) and multivariate regression (Figure S2B) analysis consistently showed that AI was the most important variable for predicting the above three topological features. In addition, significant and positive linear regressions were found between Neg and AI, while AD and Int exhibited negative regression relationships with AI.

**Figure 2:**
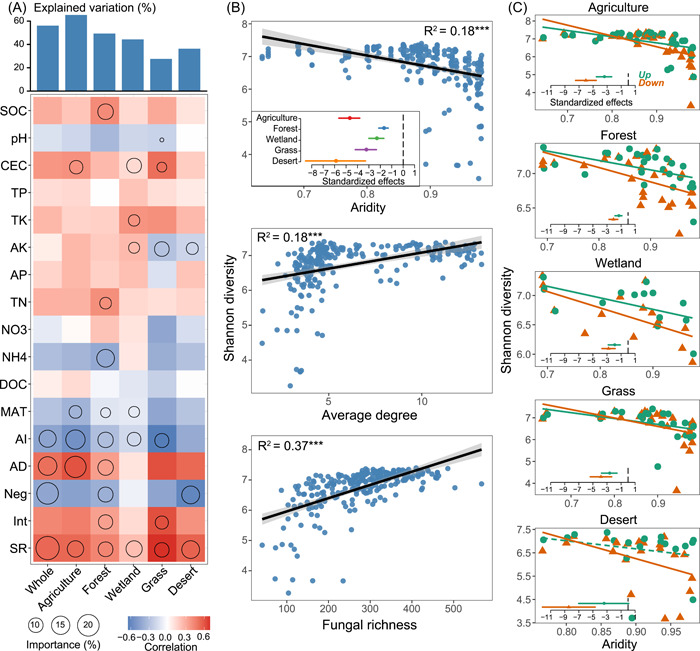
Drivers of soil bacterial α‐diversity across different biomes. (A) Contributions of abiotic and biotic factors to bacterial α‐diversity based on correlation and random forest model. Circle size represents the variables' importance (i.e., percentage of increase of mean square error calculated via random forest model). Colors represent Spearman's correlations. The abbreviations of edaphic and climatic properties accorded to the *Method*. AD, average degree; Neg, the proportion of negative associations; Int, the proportion of interacted associations between bacterial and fungal taxa; SR, soil fungal richness. (B) Relationships between bacterial α‐diversity and the main drivers were estimated via linear least‐squares regression analysis. Standardized effects (standardized slopes [mean ± SEM]) of aridity on bacterial α‐diversity were compared among different biomes. (C) Relationships between aridity and the bacterial α‐diversity in surface (0−15 cm) and subsurface (15–30 cm) layers, estimated via linear least‐squares regression analysis. Standardized effects of aridity on bacterial α‐diversity were compared between these two layers

Across all abiotic (e.g., edaphic and climatic) and biotic factors, fungal richness, AD and AI contributed the most toward explaining variation in bacterial α‐diversity of different habitats (Figure 2A). This result was confirmed by significant and negative simple linear regressions found between AI and bacterial α‐diversity, whereas the bacterial α‐diversity increased with greater fungal richness and AD (Figure 2B). Specifically, the strongest negative effect of AI on bacterial α‐diversity was observed in desert soils, followed by agricultural soils; while the effect of AI on bacterial α‐diversity was the weakest in forest soils (Figure 2B). In addition, the effect of AI on bacterial α‐diversity was stronger in the subsurface than in surface layers of all habitats (Figure 2C), suggesting that bacterial α‐diversity was more affected by AI at the subsurface layer irrespective of different habitats.

### Soil bacterial *β*‐diversity patterns along the Hexi Corridor

**Figure 3:**
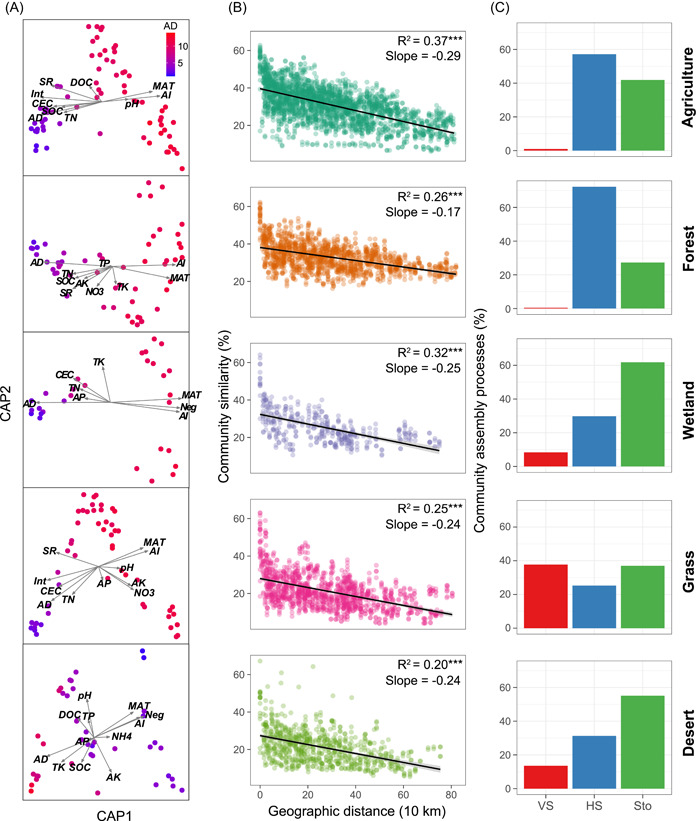
Biogeographic patterns of bacterial β‐diversity across different biomes. (A) Constrained analysis of principal coordinates (CAP) showing abiotic and biotic factors that influenced bacterial assembly. Sample points are colored according to the average degree (AD). (B) Distance–decay curves showing Bray–Curtis similarity against geographic distances between sampling sites. Solid lines denote the ordinary least squares linear regressions. Asterisks denote significant correlation (***p < 0.001). (C) The fraction of turnover in the assembly of soil bacterial communities, as governed primarily by deterministic (homogeneous [HS] and variable selection [VS]) and stochastic processes (Sto)

**Figure 4:**
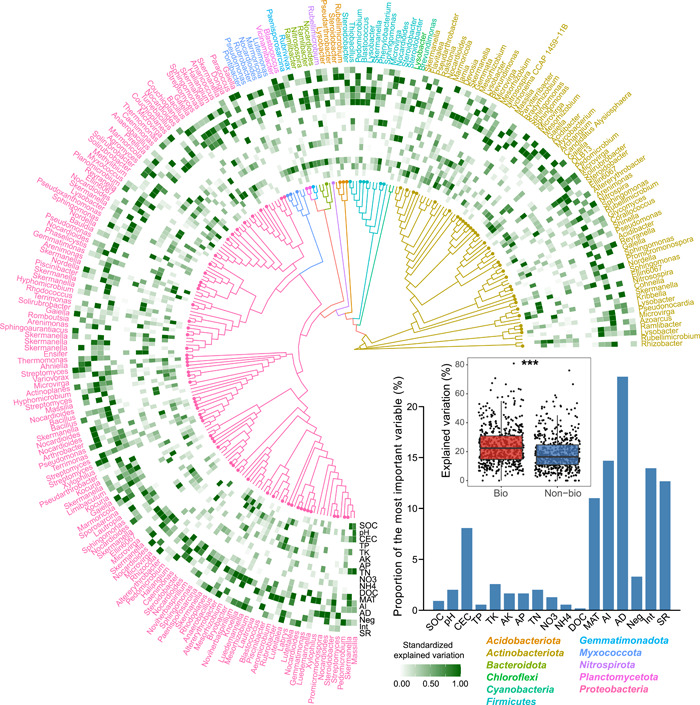
Phylogenetic distributions for the dominant bacterial taxa, and their main drivers. Highly abundant (top 10% in terms of relative abundance) and ubiquitous (presenting in all soil samples) ASVs were selected in this analysis. The phylogenetic tree was constructed using the neighbor‐joining method. The heatmap shows the relative importance of abiotic and biotic factors in explaining the selected dominant taxa, estimated via multiple regression modeling and variance decomposition analysis. Proportions of the most important variables explaining the variation in dominant taxon abundance are shown as a barplot. Boxplots show the differences in the explained variations between considering the biotic and abiotic factors (Bio) and only considering the abiotic factors (Non‐bio). ASV, amplicon sequence variant

The major abiotic and biotic factors to the bacterial β‐diversity were further identified using the constrained analysis of principal coordinates (CAP). Of the most important environmental factors contributing to the β‐diversity, AI and AD had the largest observed effect (Figure 3A, and Tables S1–S5). The contributions of environmental, geographic, and biotic variables to the variation in bacterial β‐diversity were quantified by Mantel and partial‐Mantel tests. Biotic factors were better predictors of bacterial β‐diversity than environmental and geographic ones (Table S6), indicating a stronger effect of biotic interactions in driving the β‐diversity of soil bacteria. To examine whether biotic factors played major roles in determining the distributions of specific members in the community, we combined multiple regression modeling and variance decomposition analysis to quantify the contributions of the factors to each dominant taxon, which were ubiquitous (>50% of all samples) and abundant (the top 10% in terms of relative abundance). The results showed that AD was the most frequent of the best predictors of their abundance, followed by AI (Figure 4). In addition, significantly more explained variations were observed when considering the biotic factors rather than only considering the abiotic factors, indicating the major roles of biotic factors in governing the assembly of specific bacterial members.

### Soil bacterial community assembly and their influencing factor

**Figure 5:**
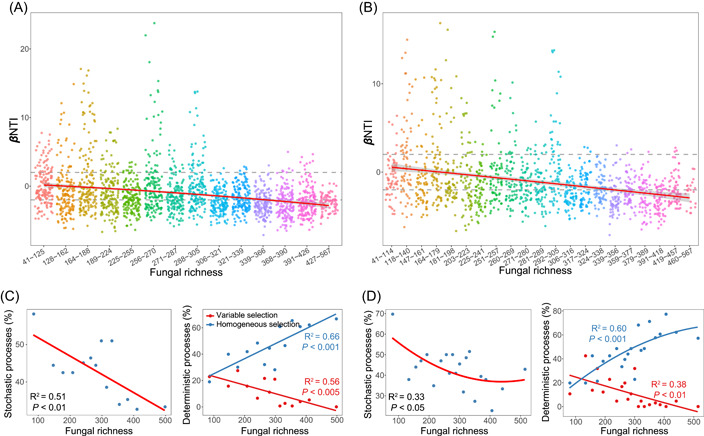
Relative influence of soil fungal richness on the deterministic and stochastic assembly processes in shaping soil bacterial communities. (A,B). Patterns of *β*NTI across the different categories of fungal richness, based on artificially separating into 14 (A) and 21 groups (B). (C,D) Horizontal dashed lines indicate the *β*NTI significance thresholds of +2 and −2. Relationships between assembly processes and fungal richness based on separating into 14 (C) and 21 groups (D), which were estimated via linear least‐squares regression analysis with second‐order polynomial fits

Community similarity versus geographic distance for each pairwise set of samples clearly displayed a significant DDR for bacterial communities (Figure 3B). However, the slopes of distance–decay that were estimated by linear regression models varied across different habitats. The slope in the agricultural soils (slope = −0.37) was significantly steeper than the other habitats, and the slope in forest soils was the flattest (slope = −0.17). We then estimated the community assembly processes behind these DDRs. Null model analyses revealed that the deterministic assembly was dominant (>50%) in bacterial communities in agricultural, forest, and grass soils, whereas stochastic assembly contributed a larger fraction to the assembly of bacterial communities in wetland and desert soils (Figure 3C). In particular, the assembly of bacterial communities in agricultural and forest soil was governed by the homogeneous selection, and variable selection contributed to a larger fraction in grasslands. The relationships between *β*NTI and major factors were used to infer changes in the relative influences of deterministic and stochastic assembly processes. The Mantel tests results showed fungal richness to be the best predictor of *β*NTI (Table S7), as evinced by the pairwise comparisons of *β*NTI values with differences in fungal richness (Figure 5A,B). With increasing in fungal richness, the relative influence of stochastic assembly processes of bacterial communities decreased, and that of homogeneous and variable selection, respectively, increased and decreased (Figure 5C,D).

**Figure 6:**
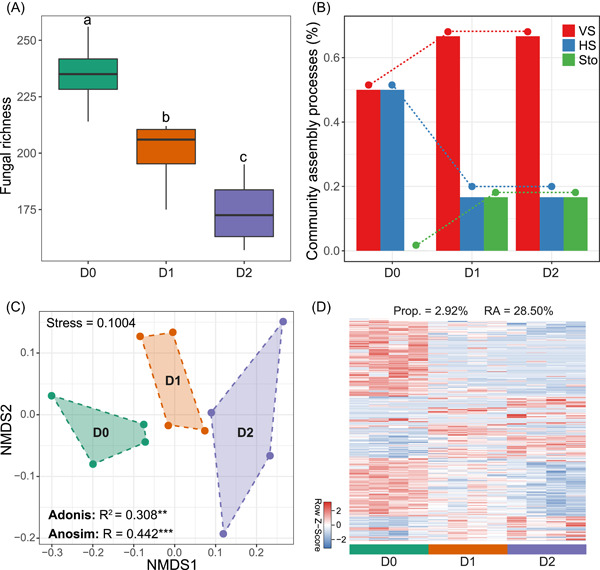
Linkages between soil fungal richness and bacterial community assembly in a microcosm study. (A) Difference in soil fungal richness among treatments with different fungicide concentrations (D0, no addition; D1, 6 mg kg^−1^; D2, 14 mg kg^−1^). Data that do not share a letter are significantly different between treatments (*p* < 0.05; multiple comparisons with Kruskal–Wallis tests). (B) Variations in assembly processes of bacterial communities among treatments, estimated via deterministic (homogeneous [HS] and variable selection [VS]) and stochastic processes (Sto). (C) Nonmetric multidimensional scaling (NMDS) ordination plot showing the bacterial β‐diversity among treatments. The significances were examined using the Adonis and Anosim tests (**p < 0.01; ***p < 0.001). (D) Heatmap of the bacterial taxa showing significant differences in relative abundance among treatments. Each row in the heatmap has been standardized (to a mean of zero and a standard deviation of one) with its color intensity proportional to the standardized relative abundance of the taxa. Prop., the proportions of the differentiated taxa; RA, the total abundance of the differentiated taxa

To provide a further test of the importance of fungal richness for bacterial community assembly, we conducted a manipulative microcosm experiment with independent soil samples, at the local stand level. Our goal was to experimentally create a gradient of soil fungal richness by using fungicide in independent soil collected from grassland in northwest China. In this microcosm, we assessed the influence of soil fungal richness on bacterial community assembly processes and the variations of specific bacterial taxa. We found fungal richness significantly decreased with the increase of fungicide concentrations (Figure 6A), indicating the experimental success in the generation of fungal richness gradient. In this context, the relative influence of stochastic assembly processes of the bacterial communities increased, and homogeneous and variable selection respectively decreased and increased along with a decrease in fungal richness (Figure 6B), consistent with the above observations based on large‐scale survey (Figure 5). In addition, bacterial β‐diversity significantly differed among the fungal richness gradients (Figure 6C), and there were substantial specific bacterial taxa that significantly varied along the gradients (Figure 6D). While no significant difference was observed in bacterial α‐diversity among fungal richness gradients (Figure S3). The results from this microcosm study provided independent and experimental verification of the important roles of fungal richness in mediating the balance of assembly processes of soil bacterial communities.

## DISCUSSION

Revealing the assembly mechanisms of belowground microbial communities is a key topic in ecology [6], which have been explored extensively in microbial ecology [7]. Few studies have mapped imprints of species associations on changes of microbial β‐diversity [76]. Here we explored the underlying assembly processes of soil bacterial community across different habitats and regions along an oasis–desert ecotone in northwest China, by considering the biotic factors inferred from soil fungi and their species associations. We provide solid evidence—from a large‐scale survey and a microcosm experiment—for the major roles of biotic factors (i.e., soil fungi and cross‐kingdom species associations) in shaping the bacterial landscapes and mediating the community assembly. In particular, soil fungal richness mediated the balance of assembly processes of soil bacterial communities, with stochastic assembly processes decreasing with an increase in fungal richness. Beyond fungal richness, aridity was the most important abiotic factor in influencing the bacterial α‐diversity, with more negative effects in desert soils and subsurface layer.

Aridity is increasing worldwide because of climate change and could substantially influence the structure and functioning of dryland ecosystems [77, 78, 79]. In the present study, we observed that across all abiotic (e.g., edaphic and climatic) factors, aridity contributed the most toward explaining variation in bacterial α‐diversity of different habitats in our sampling arid regions, and increases in aridity were linearly associated with reductions in bacterial α‐diversity. This is supported by a previous study, reporting similar trend in global drylands [78]. Specifically, the bacterial α‐diversity in desert soils was the most negatively affected by aridity comparing with other habitats, suggesting that desertification could aggravate the loss of soil microbial diversity under the increase of global aridity. Desertification is a serious problem, leading to the land degradation, biodiversity loss and functioning reducing of dryland ecosystems [80]. In addition, the negative effect of AI on bacterial α‐diversity was stronger in the subsurface than in surface layers of all habitats, implying that the losses of microbial diversity in deeper soils are more dramatic to the increase of aridity such as those forecasted with climate change. Given that subsoils contain >50% of global soil organic carbon stocks and nearly 35% of the total microbial biomass [81, 82], the losses of biodiversity in deeper soil might substantially influence the carbon cycling of the terrestrial ecosystem. Previous study showed that aridification led to systemic and abrupt changes in multiple ecosystem attributes, and more than 20% of the terrestrial surface will cross the aridity thresholds by 2100 [77]. Uniquely, our results suggest that the policies developed to minimize the negative impacts of aridification on belowground biodiversity and functions in terrestrial ecosystem should not overlook the land‐use change and sensitive subsurface soils.

We also found that more frequent co‐occurrences (higher degree) among microbial taxa in the low‐aridity environments, supported by the notion that higher precipitation strengthens microbe–microbe interactions [83]. Higher network complexity under a lower level of aridity might be partially explained by the increasing biomass stimulated by a greater supply of water, providing more opportunities for different species to interact with each other [83]. In addition, significant and positive linear regressions were observed between negative microbial associations and aridity, indicating that an increase in aridity might induce more antagonistic or competitive biological interactions. This might be the result of the low moisture in high‐aridity environments leading to lower community stability with more competition between species [84], which could reduce the efficiency of resource transfer compared with those inhabiting a more isolated space [85]. On the basis of the above findings, we revealed that aridity is an important abiotic factor in shaping the bacterial community structure and species coexistence.

Species interactions, which could determine the functional attributes or niche occupancy of microbial communities [30, 31], play important roles in stimulating ecosystem processes [47]. Previous studies demonstrated that species association played important roles in driving β‐diversity of diazotrophic and bacterial communities in paddy soil [26]. In the present study, we revealed that biotic factors were better predictors of bacterial α‐ and β‐diversity compared with environmental ones, indicating a larger role for biotic interactions in driving the diversity and assembly of soil bacterial communities across different habitats. This observation was also confirmed by examining the major roles of biotic factors in determining the distributions of specific members in the communities. The biotic mechanisms were probably involved with species association, which is typically used in ecology and biogeography as a proxy for species interaction in the community [26, 86]. Species interactions due to fitness differences, for example, competition and mutualisms, could result in the niche partitioning of community members under environmental heterogeneity [33, 34]. For example, competition caused by limited nutrient sources and antagonistic effects among species has been suggested to limit the coexistence of species [30, 44, 45, 46], which has consequences for microbial community assembly [27]. Metabolic interdependence among taxa could induce species coexistence that leads to aggregation of microbes [87]. Thus, these studies could support our conclusion that biotic interactions between microbial taxa contribute substantial variations in community β‐diversity across different habitats at large scales, corresponding to our first hypothesis.

Disentangling the assembly mechanisms of belowground microbial communities is crucial to better understand the maintenance and generation of terrestrial microbial diversity [6]. In our study, pairwise comparisons of *β*NTI values of bacterial communities were significantly correlated with differences in fungal richness, suggesting that fungal richness was closely linked to the balance between stochastic and deterministic assembly processes of soil bacterial communities. Our experimental tests further support the observed linkages between soil fungal richness and mechanisms underlying bacterial community assembly across terrestrial ecosystems using laboratory manipulations, which held most environmental sources of variation relatively constant. This is corresponding to our second hypothesis. In particular, with increasing fungal richness, the relative influence of stochastic assembly processes of bacterial communities decreased, and that of homogeneous and variable selection respectively increased and decreased. This could be due to the complex interaction between fungi and bacteria in the soil. For example, some soil‐derived fungal species could synthesize antibiotics and showed antagonistic effects on bacterial species [88, 89] or soil fungi could decompose the recalcitrant organic matter, e.g. cellulose and lignin, providing the substrates for bacterial symbiotically utilization [49]. Given these cases, higher fungal richness might induce more homogeneous sorting effects on bacterial taxa, resulting in their weaker stochastic assembly and stronger homogeneous selection. Together, our study—to our knowledge—first built the linkage between soil fungal richness and mechanisms underlying bacterial community assembly, and highlighted the potential roles of cross‐kingdom biotic interactions in regulating the balance of microbial community assembly processes across complex terrestrial ecosystems.

A few potential limitations should be considered within the context of the present study. First, some biotic factors were inferred from the topological properties of the cross‐kingdoms network. Correlation network analyses are only a simplistic representation of a complex system. In addition, ecological networks that are based on correlations can yield spurious results [90], and associations between taxa within such networks cannot be automatically interpreted as interactions. However, ecological network information is still essential for offering insights into the topological properties of community members [35, 36, 37, 63, 64] and is regarded as a valuable tool to identify species associations within a community [26, 39, 91]. Second, fungicide cyclohexemide might be toxic for all eukaryotic organisms and therefore other groups rather than fungi were probably affected. Cyclohexamide might only be acting on a subset of the fungi as well, potentially selecting fungal members that interact more favorably/significantly with the bacteria. Fungicide could also be a resource for bacteria, and the setup of pot experiments might limit the immigration of bacteria; these could affect the bacterial community assembly. More elaborate experiments under natural conditions would be conducted in future work.

## CONCLUSIONS

Our findings provide observational and experimental evidence to reveal the major roles of biotic factors and aridity in shaping the bacterial landscapes and mediating the community assembly in complex terrestrial ecosystems. Specifically, soil fungal richness mediates the balance of assembly processes of soil bacterial communities, with stochastic assembly processes decreasing along with an increase in fungal richness; this is true in both our cross‐biomes study and manipulated experiment. Increased aridity conditions due to climate change could reduce the bacterial α‐diversity, with more negative effects in desert soils and subsurface layers, and induce more antagonistic or competitive biological interactions. Together, our research represents an important step to link soil fungi to the mechanisms underlying biogeographic patterns and community assembly of soil bacteria in arid terrestrial ecosystems. Considering the importance of cross‐kingdom biotic interactions for the community assembly, future empirical and theoretical research are needed to investigate the biotic mechanisms underpinning the generation and maintenance of microbial diversity in response to future climate aridity changes, which together with other adverse effects (e.g., reduced water availability) may pose serious threats to key ecological processes and services, such as food production, in drylands worldwide.

## CONFLICT OF INTERESTS

The authors declare that there are no conflict of interests.

## AUTHOR CONTRIBUTIONS

Shuo Jiao conceived and designed the study with the help of Gehong Wei and Weimin Chen, Shuo Jiao, and Baogang Zhang performed the experiments; Shuo Jiao analyzed the data and drafted the manuscript; Haiyan Chu reviewed and revised the manuscript.

## Supporting information

Supporting information.

Supporting information.

## Data Availability

The scripts used in this study can be found at https://github.com/shuojiao/Soil-fungi-drive-bacterial-community-assembly. The raw sequence data reported in this paper have been deposited in the Genome Sequence Archive [92] in BIG Data Center, Beijing Institute of Genomics (BIG), Chinese Academy of Sciences, under accession numbers PRJCA004036 and PRJCA004037 that are publicly accessible at https://ngdc.cncb.ac.cn/gsa.
