# Supplementary material for: Linking soil fungi to bacterial community assembly in arid ecosystems
Source: Imeta. 2022 Feb 24;1(1):e2. doi: 10.1002/imt2.2 (PMC10989902; doi:10.1002/imt2.2)
Supplement: Supplementary file 1 — Supporting information. [file IMT2-1-e2-s002.docx]

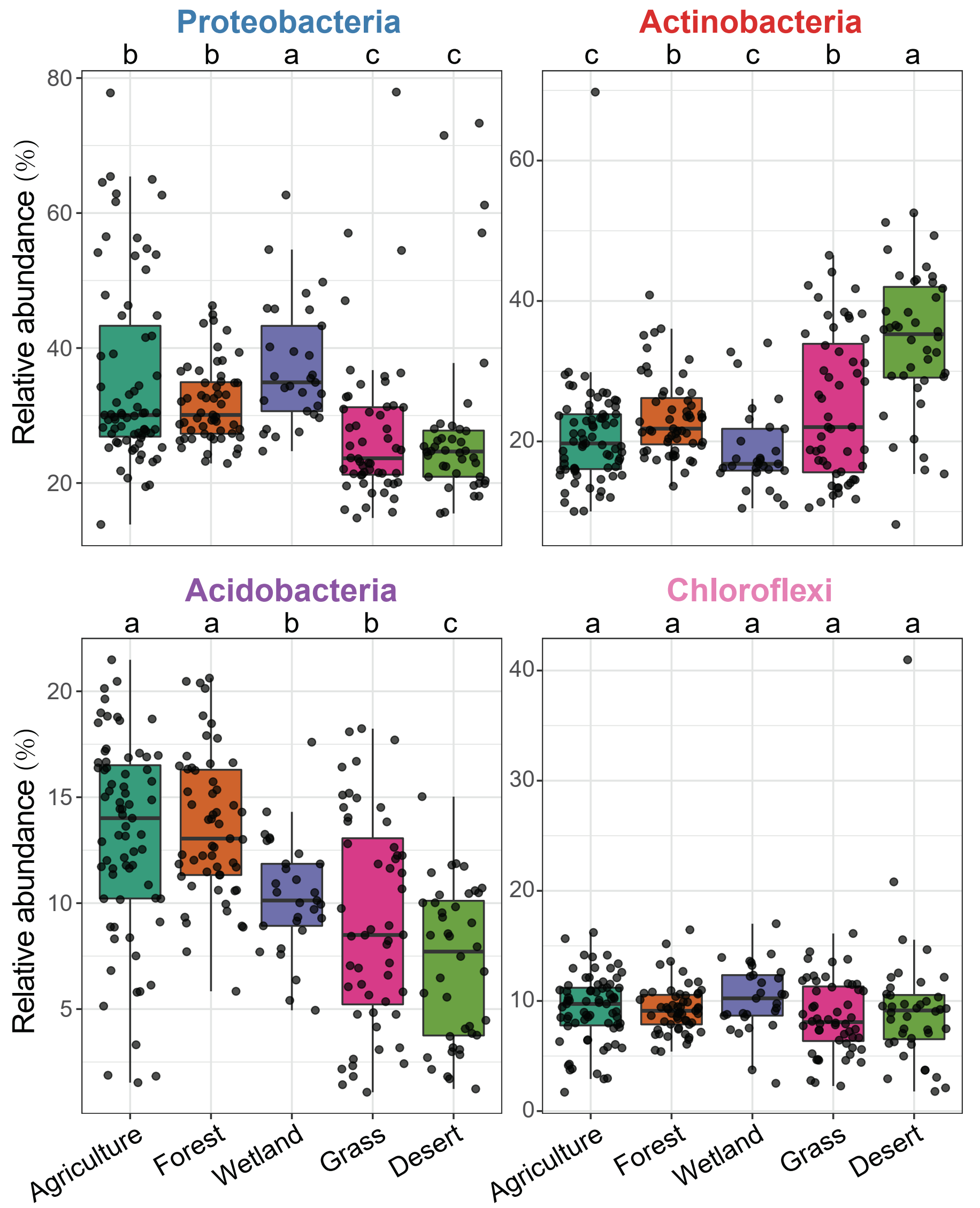


**Figure S1.** Difference in the relative abundance of major phyla among different biomes. Data that do not share a letter are significantly different between biomes (*P* <0.05; multiple comparison with Kruskal-Wallis tests).


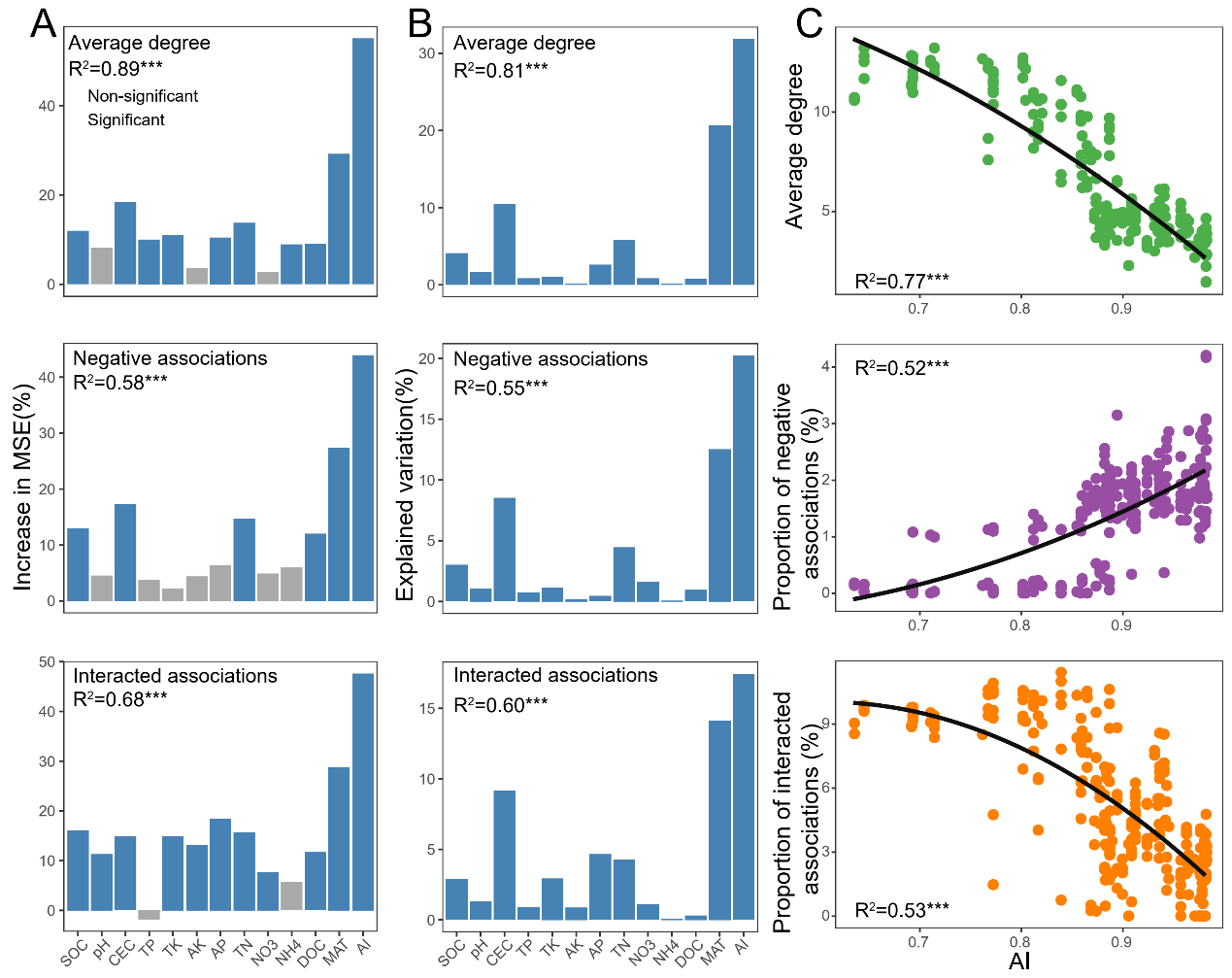
**Figure S2.** Drivers for the topological features of the sub-networks in each soil sample. **A.** Random Forest mean predictor importance of influencing factors for the topological features. The accuracy importance measure was computed for each tree and averaged over the forest (5000 trees). Percentage increases in the MSE (mean squared error) of variables was used to estimate the importance of these predictors, and higher MSE% values implied more important predictors. **B.** Explained variation of influencing factors for the topological features estimated via multiple regression modeling and variance decomposition analysis. **C.** Relationships between aridity and the topological features were estimated via linear least-squares regression analysis with second-order polynomial fits. *P* values were indicated by asterisks: ****P* < 0.001.


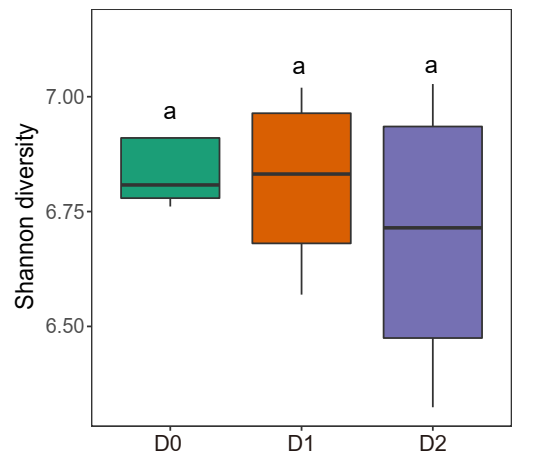
**Figure S3.** Difference in soil bacterial *α*-diversity among treaments with different fungicide concentrations (D0, no addition; D1, 6 mg kg^-1^; D2, 14 mg kg^-1^) in the microcosm study. There were no significant difference in soil bacterial *α*-diversity between treatments (*P* > 0.05; multiple comparison with Kruskal-Wallis tests).
